# Comparative ANNs with Different Input Layers and GA-PLS Study for Simultaneous Spectrofluorimetric Determination of Melatonin and Pyridoxine HCl in the Presence of Melatonin’s Main Impurity

**DOI:** 10.3390/molecules18010974

**Published:** 2013-01-14

**Authors:** Hany W. Darwish, Mohamed I. Attia, Ali S. Abdelhameed, Amer M. Alanazi, Ahmed H. Bakheit

**Affiliations:** 1 Department of Pharmaceutical Chemistry, College of Pharmacy, King Saud University, P.O. Box 2457, Riyadh 11451, Saudi Arabia; E-Mails: asaber@ksu.edu.sa (A.S.A.); amalanazi@ksu.edu.sa (A.M.A.); abakheit@ksu.edu.sa (A.H.B.); 2 Analytical Chemistry Department, Faculty of Pharmacy, Cairo University, Kasr El-Aini St., Cairo 11562, Egypt; 3 Department of Medicinal and Pharmaceutical Chemistry, Pharmaceutical and Drug Industries Research Division, National Research Centre, Dokki, Giza 12622, Egypt

**Keywords:** melatonin, pyridoxine HCl, spectrofluorimetry, multivariate calibration methods, pharmaceutical tablets

## Abstract

Melatonin (MLT) has many health implications, therefore it is important to develop specific analytical methods for the determination of MLT in the presence of its main impurity, *N*-{2-[1-({3-[2-(acetylamino)ethyl]-5-methoxy-1*H*-indol-2-yl}methyl)-5-methoxy-1*H*-indol-3-yl]ethyl}acetaamide (DMLT) and pyridoxine HCl (PNH) as a co-formulated drug. This work describes simple, sensitive, and reliable four multivariate calibration methods, namely artificial neural network preceded by genetic algorithm (GA-ANN), principal component analysis (PCA-ANN) and wavelet transform procedures (WT-ANN) as well as partial least squares preceded by genetic algorithm (GA-PLS) for the spectrofluorimetric determination of MLT and PNH in the presence of DMLT. Analytical performance of the proposed methods was statistically validated with respect to linearity, accuracy, precision and specificity. The proposed methods were successfully applied for the assay of MLT in laboratory prepared mixtures containing up to 15% of DMLT and in commercial MLT tablets with recoveries of no less than 99.00%. No interference was observed from common pharmaceutical additives and the results compared favorably with those obtained by a reference method.

## 1. Introduction

The pineal hormone, melatonin (*N*-acetyl-5-methoxytryptamine, MLT), is a neurohormone primarily synthesized and released by vertebrates pineal gland in a circadian fashion at night. MLT has been shown to be an important hormone in modulation of several endocrinological, neurophysiological, and behavioral functions [1,2,3]. These effects result from the activation of at least two high-affinity G-protein coupled receptors, designated as MT_1_ and MT_2_, localized in the central nervous system and in peripheral tissues [4]. In human beings, MLT has been shown to be applicable in the treatment of sleep disorders to improve sleep quality [5], alleviate jet lag [1], and as an effective free radical scavenger and anti-oxidant [6].

**Figure 1:**
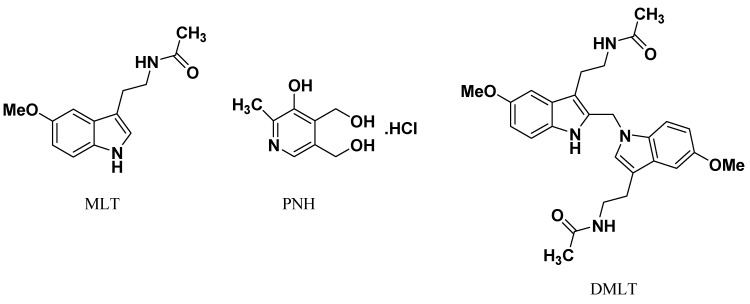
Chemical structures of melatonin (MLT), pyridoxine HCl (PNH), and DMLT.

MLT administration (15 mg/day) to cancer patients for four weeks has led to induction of eosinophilia [7,8]. It has been documented that three different commercial MLT preparations contain six contaminants structurally related to those found in contaminated L-tryptophan (Trp) preparations [9]. MLT contaminants are present in amounts of 0.1–0.5% of the parent MLT in the investigated commercial MLT preparations based on both UV and MS responses [9]. Contaminants in Trp preparations are responsible for the 1989 outbreak of eosinophilia-myalgia syndrome (EMS), which affected about 1,500 people and led to about 30 deaths in the United States [10]. Two contaminants in commercial MLT preparations were identified to be hydroxymelatonin isomers with *m*/*z* [M+H]^+^ = 249 whereas, other four contaminants were identified as melatonin-formaldehyde condensation products with *m*/*z* [M+H]^+^ = 477 [9]. DMLT (Figure 1) is the most abundant regioisomer among the four melatonin-formaldehyde condensation contaminants that were found in the commercial preparations of MLT [9].

Pyridoxine hydrochloride (PNH, Figure 1), as one form of vitamin B_6_, is an essential vitamin for humans. PNH is a water soluble vitamin and involved principally in amino acid, carbohydrate and fat metabolism. It is required for the formation of hemoglobin as well as many different physiological properties [11,12]. Additionally, PNH is co-formulated with MLT in many dietary supplements for health-care purposes and sold over-the-counter in the United States.

An evaluation of the literature revealed that a HPLC/tandem mass spectrometry (LC/MS/MS) method [9] in addition to our spectrofluorimetric methods [13] have been published for the determination of only MLT in the presence of DMLT. Other studies showed that MLT alone was determined by liquid chromatography (LC) with electrochemical detection [14,15,16,17,18], fluorometric detection [16,19,20,21,22] and mass spectrometric detection [23]. Moreover, gas chromatography [24], radioimmunoassy (RIA) [25] and anodic stripping voltammetry [26] were also found in the literature for the analysis of MLT. The aforementioned techniques were utilized to analyze MLT in biological matrices such as blood, urine, pineal glands, *etc.* Additionally, a number of reports about the analytical methods for PNH were mainly LC [27,28,29] and capillary electrophoresis [30,31,32] based. There are few methods available for simultaneous measurements of MLT and PNH, such as gas chromatography-mass spectrometry [33], capillary electrophoresis with electrochemical detection [34] and zero-crossing derivative spectrophotometry [35], but they suffer from sophistication, laborious treatments and lower sensitivity, respectively. Examination of the literature exposed two chemometric methods for simultaneous determination of MLT and PNH [36,37].

A sizable number of MLT dietary supplement tablets contain PNH as a co-formulated ingredient and are sold over-the-counter. The association of MLT with PNH in many pharmaceutical preparations is probably due to the synergetic effect in the therapy of some diseases [36]. A spectrofluorimetric method for determination of MLT in the presence of its main impurity, DMLT, was recently reported [13]. An examination of the literature revealed that there are no reports of analysis of MLT and PNH in the presence of DMLT. Therefore, development of simple, accurate, and sensitive methods for the routine analysis of MLT and PNH in the presence of DMLT is of invaluable importance as daily intake of MLT is not established yet. That necessitated the development of simple and fast procedures that could be applied in quality control laboratories for the determination of MLT in presence of PNH and DMLT. So far, we have not found any reports on the simultaneous determination of MLT in presence of PNH and DMLT in dosage forms. Multivariate calibration methods such as partial least squares (PLS) and artificial neural networks (ANNs) are useful means of resolving different overlapping spectra and eliminating matrix interference in the assay of various multi-component mixtures [38,39]. The principle advantages of applying these methods to spectrofluorimetric data are; the improvement of sensitivity and selectivity as well as the significant economic advantages over other sophisticated instrumental techniques such as HPLC/tandem mass spectrometry. According to the aforementioned premises and as a continuation of our effort to develop new spectrofluorimetric methods for determination of MLT in the presence of DMLT, we report herein novel analytical methods for determination of MLT and its co-formulated interferent, PNH, in the presence of DMLT.

### 1.1. Overview of Multivariate Calibration Methods

Four chemometric methods, namely artificial neural network preceded by genetic algorithm (GA-ANN), principal component analysis (PCA-ANN) and wavelet transform procedures (WT-ANN) as well as partial least squares preceded by genetic algorithm (GA-PLS) were applied in this study. In general, this study was primarily designed to promote the proposed multivariate methods as attractive candidates for usual univariate calibration methods for fluorescence spectral data. Secondly, to make a comparative study between different variable selection and data compression procedures and their effect on increasing predictive power of PLS and ANN models. Finally, to analyze MLT and PNH in the presence of DMLT in pharmaceutical preparations since all articles, to the best of our knowledge, analyze only MLT and PNH.

#### 1.1.1. Pre-Processing Procedures

##### 1.1.1.1. Genetic Algorithm

Genetic algorithms (GA) [40,41,42,43] have been used to solve difficult problems with objective functions that do not possess ‘nice’ properties such as continuity, differentiability, *etc.* These algorithms maintain and manipulate a family, or population, of solutions and implement a ‘survival of fittest’ strategy in their search for better solutions.

###### Optimisation of Genetic Algorithm Parameters

A critical issue of successful GA performance is the adjustment of GA parameters. The parameters are: the maximum number of generations, the number of wavelengths in a window, percent genes included at initiation, the mutation rate, breeding cross over rule and percent of population the same at convergence. Other parameters to be chosen by the user are: maximum number of latent variables for the PLS, cross validation type random or contiguous blocks, number of subsets to divide data for cross validation, number of iterations for cross validation at each generation. 

##### 1.1.1.2. Wavelet Transform

WT is a recent signal processing technique [44]. Many applications of WT in chemistry appeared in the literature such as resolution of overlapped chromatographic peaks [45], signal denoising [46] and multivariate calibration [47,48].

###### Theory and Algorithm

Wavelet transform (WT) represents a windowing technique with variable-sized regions. It is similar to Short-Time Fourier Transform (STFT) in that both techniques analyze an input signal in blocks by translation (movement) of a basis function. This basis function in STFT is sine wave and it is called wavelet in WT. Wavelet analysis is the breaking up of a signal into shifted and scaled versions of the original (or mother) wavelet. Scaling a wavelet simply means stretching (or compressing) it and shifting a wavelet simply means delaying (or hastening) its onset. The WT is expressed as:

(1) $$WT_{ab}\left(t\right)=\int_{+\infty }^{-\infty }x(t)\psi [t-b/a]\;$$

where *WT _ab_* (t) is the transformed signal, *x (t)* is the original signal, *ψ* is the mother wavelet, t is time (or any pseudo-index, e.g., wavelength in spectroscopy), *a* is the scale parameter and *b* is the translation parameter. The transformed signal is a function of two variables, *b* and *a*, the translation and the scale parameters, respectively. The term translation is related to the location of the window, as the window is shifted through the signal. The translation term corresponds to time information in the transform domain. The scale parameter is defined as 1/frequency. Low frequencies (high scale) correspond to a global information of a signal (that usually spans the entire signal), where high frequencies (low scales) correspond to a detailed information of a hidden pattern in the signal (that usually lasts a relatively short time) [49].

The discrete wavelet transform (DWT) is easier to implement than the continuous wavelet transform (CWT). The CWT is computed at every possible scale while in DWT, the scale is chosen based on powers of two so called dyadic scales. An efficient way to implement DWT is the Mallat algorithm. In this algorithm, at each scale, the signal is passed through a series of high pass filters to analyze the high frequency components and passed through a series of low pass filters to analyze the low frequency components. The output from the high pass filter at each scale is recorded as the wavelet coefficients. The low pass filter extracts the low frequency components which are subjected, in the next scale; to a new set of high and low pass filters. This operation will divide the input signal into two parts, the approximation and details. The approximations are the high scale, low frequency components of the signal. The details are the low scale, high frequency components (mostly noise) that can be discarded without any major loss of the information, allowing signal compression. At each successive scale (n-1) the length of the vector upon which the filters operate is halved; this is referred to as decimation.

Many of the detailed coefficients are very small in amplitude and can be removed without major loss in the information content of the signal. There are many methods to determine the threshold value below which the wavelet coefficients can be removed safely [49]. The threshold defined by the root mean square error (RMSE) of the reconstructed signal is the most commonly used method because RMSE is a measure of the quality of compression [49]. A large error means that a significant portion of the useful information of the signal is lost after compression, while an excessively small error will affect the compression efficiency. In order to obtain optimal filter and resolution level *j* for the spectrum, the RMSE between the original measured spectrum and reconstructed signal by different wavelet filters and different resolution level *j* were investigated. 

#### 1.1.2. Multivariate Calibration Models

##### 1.1.2.1. Partial least squares regression (PLS)

PLS method involves the decomposition of the experimental data, such as spectrofluorimetric data in our case, into systematic variations (latent variables) that explain the observed variance in data. The purpose of PLS method is to build a calibration model between the concentration of the analytes under study (MLT and PNH in our case) and the latent variables of the data matrix. PLS performs the decomposition using both spectrum data matrix and analyte concentration [50]. Including extra latent variables in the model increases the possibility of the known problem of overfitting. Therefore optimization of number of the latent variables is a critical issue in the PLS method.

###### Optimisation of Number of Latent Variables for PLS Model

Cross validation (CV) [51] is applied to predict how many are the optimum number of PLS latent variables. CV involves repeatedly dividing the data into two sets, a training set used to determine a model and a test set to determine how well the model performs so that each sample (or portion of the data) is left out of the training set once only.

Leave one out (LOO) CV is used in our study for optimizing the number of PLS components, by building the model using I-1 samples set (training set consisting of 14 samples) to predict the one sample left (validation sample). The root mean square error of CV (RMSECV) is calculated as:

(2) $$\mathrm{RMSECV}=\sqrt{\frac{1}{I}{\sum_{i=1}^{I}\left(c_{i}-{\hat{c}}_{i\_cv}^{A}\right)}^{2}}$$

where *I* is the number of objects in the calibration set, *c_i_* is the known concentration for sample *i* and ${\hat{c}}_{i\_cv}^{A}$ is the predicted concentration of sample *i* using *A* components. Mean centering is performed on the training set each time successive samples are left out. In this study, PLS model preceded by genetic algorithm as variable selection procedure is applied. 

##### 1.1.2.2. Neural Networks

Artificial neural network (ANN) is a type of artificial intelligence method that resembles the biological nervous system in having the ability to find the relationship between inputs and outputs. ANN is composed of elements called artificial neurons that are interconnected by connections called weights. Commonly neural networks are trained, so that a particular input leads to a specific target output. The network is adjusted, based on a comparison of the output and the target, until the network output matches the target. Typically many input/target pairs are used to train a network [52]. ANN has greater superiority over other classical multivariate methods in modeling linear and non-linear relationship between variables [53,54,55].

The type of ANN used in this paper is the feed-forward model which is trained with the back propagation of errors learning algorithm. The back-propagation ANN is used in signal processing, data reduction and optimization, interpretation and prediction of spectra and calibration [53]. It is composed of three layers, an input layer in which the input data are introduced (e.g., FI in spectroflourometry). These inputs are passed to second hidden layer in which inputs are corrected and adjusted by weights and then finally passed to outer most layer (output layer) to give outputs (e.g., concentrations). The connections (weights) between layers are passed forward (from input to output layer), so it is called feed-forward ANN. The outputs (predicted concentrations) are compared with targets (actual concentrations) and the difference between them is called the error which is back propagated (and so called feed-forward ANN with the back propagation of errors learning algorithm) to the network once more to be minimized through further adjustment of weights. ANN is iterated several times in such a way till the error reaches a minimum value. In this study, ANN model preceded by genetic algorithm, principal component analysis and wavelet transform as input data reduction procedures are applied.

###### Optimization of the ANN model parameters

For proper training of the ANN model, several parameters have to be optimized. There are two transfer functions used in ANN, one between input and output of a node in the hidden layer and the other is applied in output layer. The use of these functions depends on relationship between the inputs and outputs. Tan sigmoid followed by purelin are commonly used for non-linear systems while purelin-purelin transfer functions are used for linear one (as in our case). 

Among other ANN parameters, the hidden neurons number (HNN) is related to the converging performance of the output error function during the learning process. The learning coefficient (Lc) controls the degree at which connection weights are modified during the learning phase. The learning coefficient decrease (Lcd) and learning coefficient increase (Lci) control the variation of Lc value. It varies as a function of performance of the ANN (the Lc decreases or increases with the mean square error). 

## 2. Results and Discussion

### 2.1. Analysis

**Figure 2:**
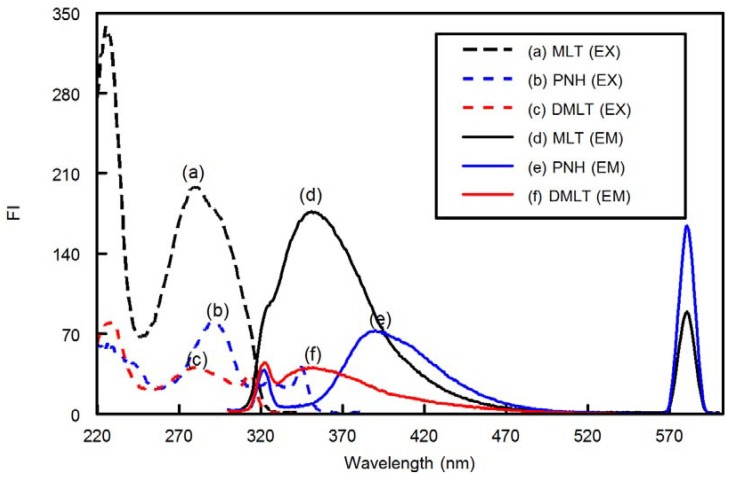
Excitation (dashed lines) and emission (solid lines) for MLT (a and d), PNH (b and e) and DMLT (c and f). Concentrations of all components were 100 ng mL^−1^ in phosphate buffer solution (pH 3, 0.1 M).

MLT, PNH and DMLT exhibited native fluorescence in methanol with λ _emission_ of 350, 390 and 350 nm for MLT, PNH, DMLT, respectively, after excitation of the compounds at 290 nm, showing great overlap in their emission spectra (Figure 2). The fact that the MLT and DMLT emission spectra are completely superimposed and that of PNH is greatly overlapped, hindered the determination of MLT and PNH in the presence of DMLT either directly or by any other univariate calibration methods such as derivative spectroscopy. Additionally, the other reported techniques such as chromatography, capillary electrophoresis and radioimmunoassy are more sophisticated and require laborious procedures. Therefore, application of multivariate calibration methods such as PLS and ANNs represent the most suitable analytical procedures for resolving such complex spectra. To the best of our knowledge, this is the first method for the simultaneous determination of MLT and PNH in the presence of DMLT.

#### 2.1.1. Spectral Characteristics and Optimization of Assay Conditions

Different experimental parameters affecting the emission spectra of MLT and PNH were carefully studied and optimized. Such factors were changed individually while others were kept constant. These factors included pH, buffer volume, type of the diluting solvent and stability time.

##### 2.1.1.1. Effect of pH

**Figure 3:**
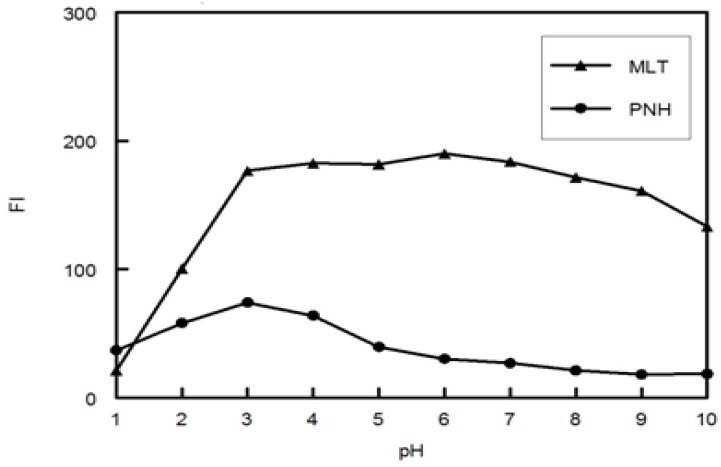
Effect of pH on FI of MLT and PNH (100 ng mL^−1^).

The influence of pH on the fluorescence intensity (FI) and fluorescence range of the MLT and PNH was studied using phosphate buffer covering the pH range from 1.0 to 10.0. It was clear from Figure 3 that phosphate buffer of pH 3.0 was suitable to give highest FI for both MLT and PNH. 

##### 2.1.1.2. Effect of Buffer Volume

**Figure 4:**
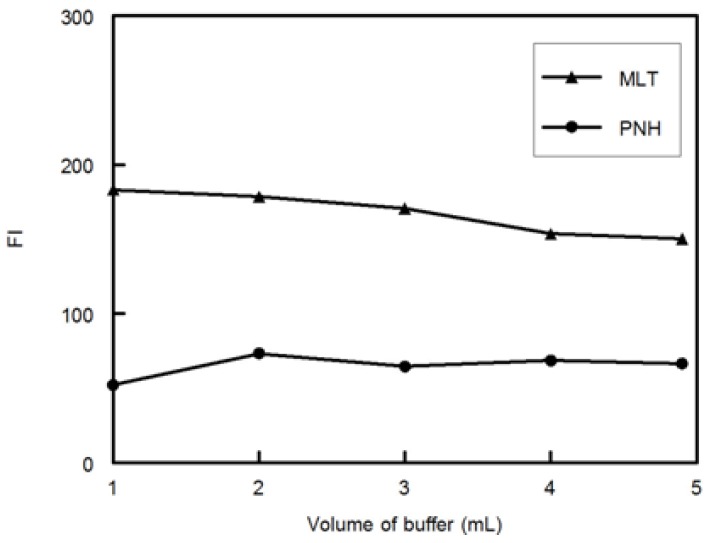
Effect of volume of phosphate buffer on FI of MLT and PNH (100 ng mL^−1^).

The effect of phosphate buffer volume on the FI of MLT was studied. Figure 4 shows that only 2 mL of phosphate buffer was sufficient to reach maximum FI for MLT and PNH.

##### 2.1.1.3. Effect of Diluting Solvent

**Figure 5:**
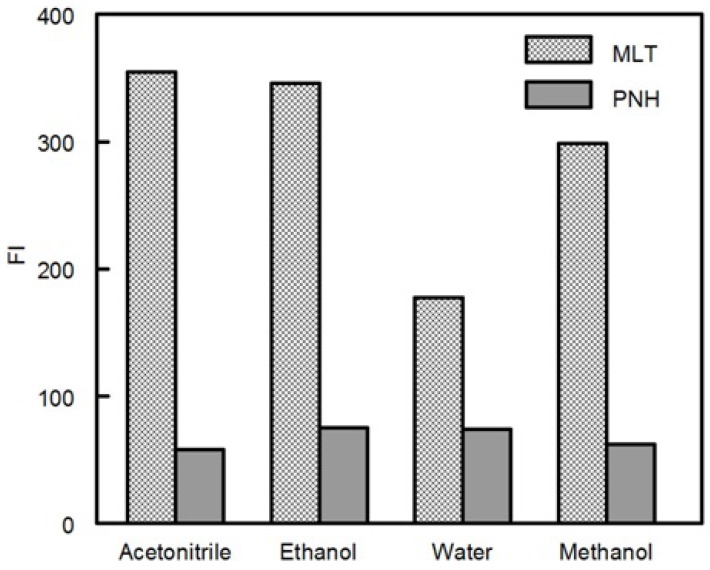
Effect of diluting solvent on FI of MLT and PNH (100 ng mL^−1^).

Dilution with different solvents including water, methanol, ethanol, and acetonitrile was employed. Figure 5 demonstrates that the effect of diluting solvent is minimal for FI of PNH, while FI of MLT varied with different solvents. Despite the fact that water did not produce the highest sensitivity, it was chosen as the diluting solvent throughout the study to allow the incorporation of the 3:1 ratio of MLT and PNH (which is the ratio found in the investigated dosage form). 

##### 2.1.1.4. Effect of Time

**Figure 6:**
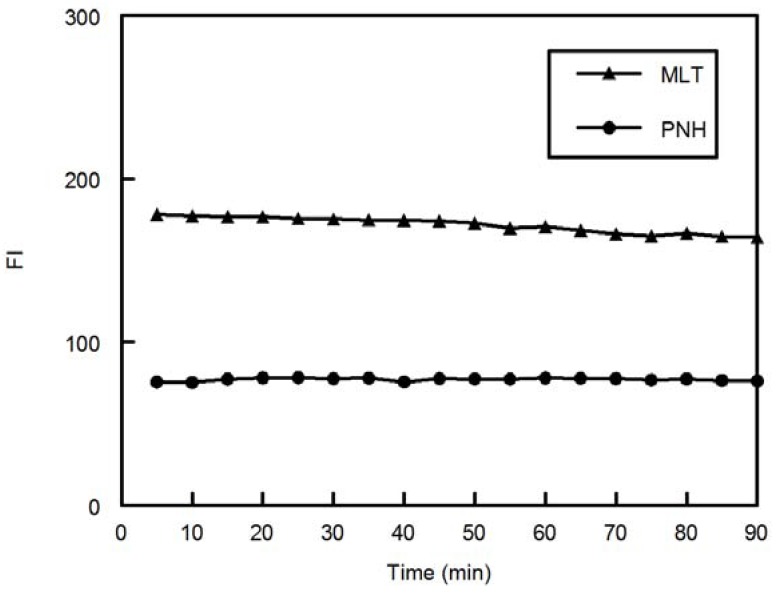
Effect of time on FI of MLT and PNH (100 ng mL^−1^).

The effect of time on the stability of the FI of the drugs was also studied. It was found that the FI developed instantaneously and remained stable for at 90 minutes (Figure 6).

#### 2.1.2. Optimization Parameters and Calibration Procedures

**Figure 7:**
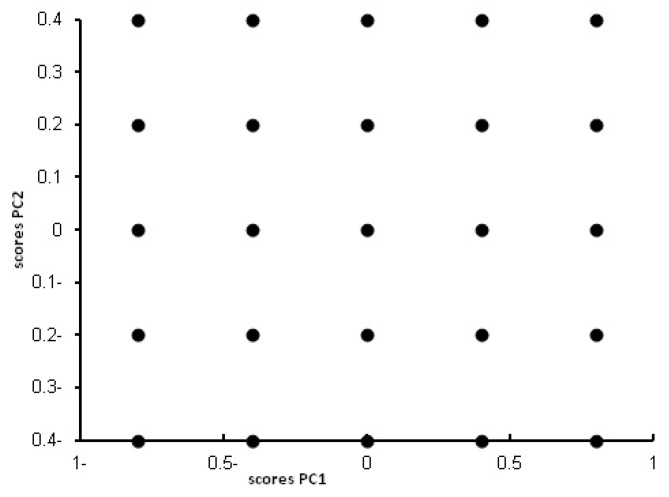
Scores plot for the mean centered 25 samples concentration matrix of the five level two component experimental design.

The purpose of multivariate methods is to build a calibration model between the concentration of the analytes under study (MLT and PNH in our case) and the experimental data (FI in our case). The first step in model building, involves constructing the calibration matrix for the ternary mixture. In this study calibration set was optimized with the aid of the five level three factor design [54] resulting in 25 sample mixtures. Table 1 shows the composition of the 25 sample mixtures. The concentration of MLT and PNH in those 25 samples was chosen according to the calibration range of each of MLT and PNH and the ratio of MLT to PNH in commercial preparations. The 2D Scores plot for the first two PCs of the whole concentration matrix was obtained to confirm the well position of the mixtures in space, orthogonality, symmetry and rotatability [56] as indicated in Figure 7. Mean centering of the data proved to be the best preprocessing method for getting the optimum results. The 25 sample mixtures were divided into 15 training mixtures (for building the models) and 10 validation mixtures (for measuring predictive power of the models).

##### 2.1.2.1. GA-PLS

For the PLS model, calibration was done by performing the decomposition of the experimental data matrix into latent variables using both the FI data matrix and the analytes concentration matrix. The emission spectra of these mixtures were collected and examined; after manipulation of data matrices, PLS method was run for optimizing the number of latent variables using leave one out (LOO) CV and RMSECV was calculated as mentioned in Section 1.1.2.1. The selection of the optimum number of latent variables was a very important pre-construction step: if the number of factors retained was more than required, more noise would be added to the data; if the number retained was too small, meaningful data that could be necessary for the calibration might be discarded. The optimum number of latent variables for PLS model was 2. 

In the GA-PLS method, the same procedures were applied for construction of the model but the PLS model was preceded by GA procedures as variable selection to choose the most correlated wavelengths to the concentrations of the analytes. A critical issue of successful GA performance is the adjustment of GA parameters. Optimum parameters for the genetic algorithm were summarized in Table 2. 

The fitness values were used as response variables for adjustment of these parameters. The GA was run for emission spectra using a PLS with maximum number of latent variables allowed. The optimal number of components determined by cross-validation on the model FI matrix is less than half for MLT (90 instead of 201 wavelengths) and about one fourth for PNH (55 instead of 201 wavelengths). The chosen wavelengths for MLT are 340–344, 370–379, 385–394, 405–424, 430–444, 450–464, 470–474, 480–484, 490–494 (totally, 90 nm) while that for PNH are 375–379, 385–389, 405–424, 435–444, 450–454, 465–474 (totally, 55 nm).

##### 2.1.2.2. ANNs

**Figure 8:**
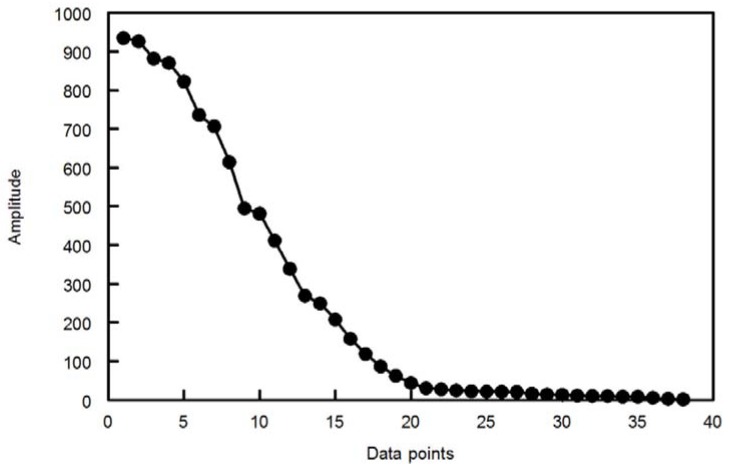
Plot of the absolute values of coefficient vector sorted by magnitude obtained by applying DWT to the simulated signal.

The second, third and fourth methods depended on the ANN approach. Since the large number of nodes in the input layer of the network (*i.e.*, the number of wavelength readings for each mixture) increases the CPU time for ANN modeling, the FI matrix was reduced either by variable selection procedure (e.g., GA) or by variable compression procedures (e.g., PC and WT). FI matrix was reduced to less than half for MLT and about one fourth for PNH in GA-ANN (as in GA-PLS), two principal components in PCA-ANN and 21 wavelet coefficients in WT-ANN. Figure 8 shows the wavelet coefficients obtained with filter 6 in the Daubechies family (db6); it can be seen clearly that there are only 21 coefficients having great absolute value. Thus by removing the other small coefficients, signal denoising and compression can be done in the wavelet domain. The denoising and compression effectiveness mainly depends on the wavelet filter and resolution level. In our work, RMSE method was used as criterion for simultaneous denoising and compression [49]. The optimal filter is defined as that for which the RMSE is a minimum. The RMSE method is applied to a single spectrum so the mean spectrum of the data was used for this purpose. Different wavelet bases at different resolution level were tested on the mean spectrum like Daubechies wavelet family, Symlmet family, and Coiflet family at different levels (as shown in Table 3 and Table 4) using compression ratio5 (CR = 5) (*i.e.*, using 20% of the total coefficients). It can be seen that RMSE reaches a minimum when resolution level is 4 using db6 as wavelet basis. Once the optimal filter and resolution level were selected, all individual spectra were transformed using this filter. Then the optimal number of the wavelet coefficients (21) was used to construct data matrix, which contains the important information. This data matrix (input layer) was used for calibration in wavelet domain, *i.e.*, no reconstruction of the signal was done. The index of the retained coefficients is kept for use with future samples.

The output layer is the concentration matrix of MLT or PNH. The hidden layer consists of just a single layer which has been considered sufficient to solve similar or more complex problems. Moreover, more hidden layers may cause over-fitting [53]. For proper modeling of ANNs, different parameters should be optimized. These parameters are summarized in Table 5. Careful optimization of transfer function pair is an important aspect to consider. Choosing of transfer function depends on the nature of data under study. In our case, purelin-purelin transfer function was implemented in our models due to linear correlation between FI and concentrations.

After optimization of parameters and architectures of the three ANNs, the training step preceded. We trained ANNs by different training functions and there is no difference in performance (*i.e.*, there is no decrease in root mean square error of prediction (RMSEP)). Levenberg–Marquardt training algorithm (TRAINLM) was thus preferred as it is time saving. To avoid overfitting, the validation set was involved in training step and ANNs stops when RMSEP of calibration set decreased and that of independent set increased.

##### 2.1.2.3. Prediction ability of the Multivariate Calibration Models

**Figure 9:**
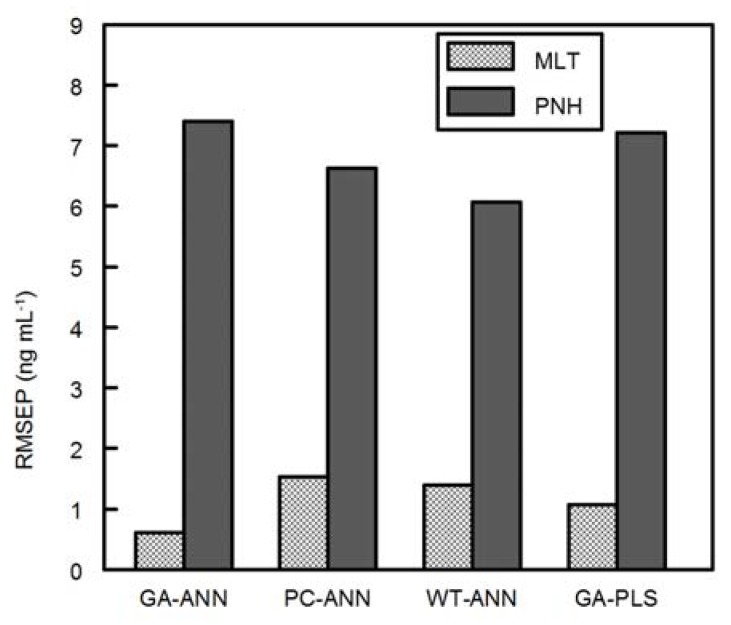
Bar plots for comparison of the RMSEP values obtained by application of the proposed multivariate calibration methods for the analysis of validation set.

After optimization of parameters and calibration (training) step, all models were applied for analysis of MLT and PNH in presence of DMLT in training set (Table 6) and in validation set (Table 5). RMSEP was calculated and used as a measure for performance of the proposed models (Figure 8) showing that the four methods predicted MLT and PNH successively in presence of DMLT. However GA-ANN is the efficient one for MLT determination as indicated by decreasing RMSEP of MLT results in validation set (Figure 9). GA-ANN showed also superiority in the determination of PNH as indicated by the low S.D. value (Table 7).

The above mentioned models were applied successfully for analysis of MLT and PNH in their combined dosage form. The results of the analysis of the MLT and PNH in bulk powder and in commercial tablets by the proposed methods were statistically compared to the reference PLS method (Table 8). The *t* and *F* values were computed and generally found to be less than the tabulated ones except in case of PCA-ANN and GA-PLS for MLT (*t* test values slightly higher than tabulated ones). 

## 3. Experimental

### 3.1. Apparatus

Fluorescence measurements were carried out on a RF-3501 version 3.0 spectrofluorimeter (Shimadzu Corporation, Kyoto, Japan) equipped with a 150 W xenon lamp and 1 cm quartz cells. The slit widths for both the excitation and emission monochromators were set at 5.0 nm. The calibration and linearity of the instrument were frequently checked with standard quinine sulphate (0.01 µg mL^─1^). Wavelength calibration was performed by measuring λ _excitation_ at 275 nm and λ _emission_ at 430 nm; no variation in the wavelength was observed. All recorded spectra converted to ASCII format by RFPC software. Hanna pH-Meter (Cluj-Napoca, Romania) was used for pH adjustments.

### 3.2. Software

All multivariate calibration methods were implemented in Matlab^®^ 7.1.0.246 (R14). GA-PLS, GA-ANN, PCA-ANN and WT-ANN were carried out by using PLS toolbox software version 2.1 in conjunction with Neural Network toolbox. The *t* test, *F* test were performed using Microsoft^®^ Excel. All calculations were performed using intel^®^ core™ i5-2400, 3.10 GHz, 4.00GB of RAM under Microsoft Windows 7.

### 3.3. Materials

MLT was obtained from AK Scientific Inc. (Union City, CA, USA). PNH was kindly donated by the Saudi Food and Drug Administration (SFDA). The purities of MLT and PNH were 99.5%. Melatonin^®^ tablets (MasoN company, Miami Lakes, FL, USA) labeled to contain 3 mg of MLT and 1 mg of PNH (Lot No: 9151J). Double distilled water was obtained through WSC-8S water purification system (Hamilton Laboratory Glass Ltd., Kent, UK) and used throughout the work. A phosphate buffer solution (pH 3, 0.1 M) was employed for pH adjustment. All solvents and materials used throughout this study were of analytical grade.

### 3.4. Synthesis

Synthesis and characterization of *N*-{2-[1-({3-[2-(acetylamino)ethyl]-5-methoxy-1*H*-indol-2-yl}methyl)-5-methoxy-1*H*-indol-3-yl]ethyl}acetamide (DMLT) and its intermediates were previously reported [13].

### 3.5. Preparation of Standard Solutions

Stock solutions of MLT (200 μg mL^−1^), PNH (200 μg mL^−1^) and DMLT (100 μg mL^−1^) were prepared by dissolving 20 mg of each of MLT and PNH in 100 mL water and 10 mg of DMLT, in 100 mL methanol. Appropriate volumes of these stock solutions were diluted with water to give working solutions of 5 μg mL^−1^ for each of MLT and PNH as well as 100 ng mL ^−1^ for DMLT. Stock and working solutions were stable for at least two weeks when stored refrigerated at 4 °C.

### 3.6. Preparation of Pharmaceutical Tablets Sample Solutions

Ten tablets of Melatonin^®^ (MasoN company) were weighed and finely powdered. An accurately weighed portion of the powder equivalent to 3 mg of MLT and 1 mg of PNH was extracted with hot water (250 mL, 80 °C for 1 h) and the extract was filtered. Aliquots of the filtrate were diluted with buffer to obtain final concentrations of 300 and 100 ng mL^−1^ for MLT and PNH, respectively, and the samples were subjected to the analysis. 

### 3.7. Calibration Procedures

Preparation of the 25 samples was carried out by transferring different volumes of MLT, PNH and DMLT from their standard working solutions into 5-mL volumetric flasks then two mL of phosphate buffer (pH 3.0, 0.1 M) was added and the solutions were diluted to the volume with water and mixed well (Table 1). 

## 4. Conclusions

Simultaneous determination of MLT and PNH in the presence of melatonin’s main impurity (DMLT) has not been reported yet. This paper shows the successful application of different multivariate calibration models for spectrofluorimetric determination of MLT and PNH in the presence of DMLT in their ternary mixtures. The multivariate methods that have been assayed are the GA-PLS, GA-ANN, PCA-ANN and WT-ANN methods. These methods are considered powerful alternatives to traditional univariate methods, especially in handling spectrofluorimetric data. Variable selection procedures (e.g., GA) as a pre-processing step showed higher efficiency than variable compression procedures (e.g., WT and PC) in increasing the predictive power of ANN. Additionally, ANN preceded by GA achieved more accuracy and precision than GA-PLS.

The proposed methods combine the rapidness and simplicity advantages of traditional spectrometric methods together with other important analytical merits, such as accuracy and specificity. The suggested methods were validated and can be applied for routine quality control analysis of MLT and PNH in their combined dosage form without prior separation and with no interference from DMLT. Additionally, sophistication and/or laborious treatments hinder the application of the other previously reported techniques, such as chromatography and capillary electrophoresis, in the routine quality control analysis for simultaneous determination of MLT and PNH in commercial preparations.

## Figures and Tables

**Table 1:** The five level three factor experimental design of the training and validation set mixtures shown as concentrations of the mixture components in ng mL^−1^.

Concentration Mixture No.	MLT ng.mL^−1^	PNH ng.mL^−1^	DMLT ng.mL^−1^	% DMLT from MLT
1	200	300	9	4.31
2	200	100	1	0.50
3	100	100	17	14.53
4	100	500	5	4.76
5	300	200	17	5.36
6	150	500	9	5.66
7	300	300	5	1.64
8	200	200	5	2.44
9	150	200	13	7.98
10	150	400	17	10.18
11	250	500	13	4.94
12	300	400	9	2.91
13	250	300	17	6.37
14	200	500	17	7.83
15	300	500	1	0.33
16	300	100	13	4.15
17	100	400	1	0.99
18	250	100	9	3.47
19	100	300	13	11.50
20	200	400	13	6.10
21	250	400	5	1.96
22	250	200	1	0.40
23	150	100	5	3.23
24	100	200	9	8.26
25	150	300	1	0.66

**Table 2:** Optimum parameters of the genetic algorithms (GA).

Parameter	Value
Population size	32
Maximum generations	40
Mutation rate	0.005
the number of variables in a window (window width)	5
per cent of population the same at convergence	80
% wavelengths used at initiation	50
Crossover type	double
Maximum number of latent variables	2
number of subsets to divide data into for cross validation	3
number of iterations for cross validation at each generation	1

**Table 3:** RMSE between experimental fluorescence spectrum of 3 components (MLT, PNH and DMLT) and reconstructed signal by DWT with different filters and resolution level J = 3 (CR = 5).

Filter	RMSE	Filter	RMSE	Filter	RMSE
Daub2	0.6474	Sym2	0.6225	Coif1	0.6796
Daub4	0.3177	Sym2	0.3828	Coif2	0.3663
Daub6	0.3078	Sym2	0.3859	Coif3	0.3886
Daub8	0.4195	Sym2	0.3673	Coif4	0.4051
Daub10	0.5390	Sym2	0.3603	Coif5	0.6425

**Table 4:** RMSE between experimental fluorescence spectrum of 3 components (MLT, PNH and DMLT) and reconstructed signal by DWT with wavelet filter Daubechies 6 and different resolution levels (CR = 5).

Resolution Level (J)	RMSE
3	0.0948
4	0.0857
5	0.1028
6	0.1496
7	0.2247

**Table 5:** Optimized parameters of ANNs.

Method	GA-ANN		PCA-ANN		WT_ANN	
Drugs	MLT	PNH	MLT	PNH	MLT	PNH
Architecture	90-4-1	55-4-1	2-2-1	2-2-1	21-5-1	21-5-1
Hidden neurons number	4	4	2	2	5	5
Transfer Functions	Purelin-purelin	Purelin-purelin	Purelin-purelin	Purelin-purelin	Purelin-purelin	Purelin-purelin
Learning coefficient	0.001	0.001	0.1	0.1	0.001	0.001
Learning coefficient decrease	0.1	0.1	0.001	0.001	0.1	0.1
Learning coefficient increase	10	10	10	10	10	10

**Table 6:** Analysis results for the prediction of MLT and PNH in the training set by the proposed multivariate calibration methods.

Method		GA-ANN				PCA-ANN				WT-ANN				GA-PLS			
MLT	PNH	MLT		PNH		MLT		PNH		MLT		PNH		MLT		PNH	
True(μg mL^−1^)		Found (μg mL^−1^)	R%	Found (μg mL^−1^)	R%	Found (μg mL^−1^)	R%	Found (μg mL^−1^)	R%	Found (μg mL^−1^)	R%	Found (μg mL^−1^)	R%	Found (μg mL^−1^)	R%	Found (μg mL^−1^)	R%
150	100	147.63	98.42	99.07	99.07	148.51	99.01	97.07	97.07	150	100.00	97.11	97.11	148.83	99.22	93.41	93.41
150	200	149.83	99.89	199.13	99.56	150.54	100.36	202.4	101.2	150	100.00	207.97	103.99	150.98	100.65	200.25	100.13
150	300	150.11	100.07	298.53	99.51	149.23	99.49	297.94	99.31	150	100.00	286.33	95.44	149.23	99.49	298.02	99.34
150	400	150.32	100.21	403.32	100.83	151.23	100.82	398.53	99.63	150	100.00	400	100.00	150.83	100.55	400.1	100.02
150	500	150.19	100.13	499.01	99.8	151.06	100.71	491.99	98.4	150	100.00	500	100.00	150.97	100.65	491.39	98.28
100	100	100.03	105.32	98.31	98.31	98.8	98.80	103.1	103.1	94.98	94.98	100	100.00	98.65	98.65	101.99	101.99
100	200	100.04	102.91	201.78	100.89	98.12	98.12	202.51	101.26	97.21	97.21	200	100.00	97.87	97.87	200.47	100.23
100	300	100.21	100.21	297.36	99.12	100.63	100.63	292.27	97.42	100	100.00	300	100.00	100.17	100.17	294.51	98.17
100	400	99.42	99.42	402.22	100.55	100.86	100.86	411.27	102.82	100	100.00	400	100.00	100.34	100.34	412.62	103.15
100	500	100.38	100.38	500.36	100.07	101.74	101.74	499.75	99.95	100	100.00	495.51	99.10	101.17	101.17	500.12	100.02
250	100	250.03	98.90	95.2	95.2	253.14	101.26	97.22	97.22	252.81	101.12	100	100.00	252.96	101.18	95.88	95.88
250	200	249.82	99.93	200.61	100.3	249.33	99.73	206.12	103.06	250	100.00	200	100.00	250.29	100.12	203.67	101.83
250	300	250.17	101.38	299.81	99.94	248.64	99.46	301.81	100.6	246.78	98.71	300	100.00	248.79	99.52	303.66	101.22
250	400	248.16	99.26	409.31	102.33	247.27	98.91	412.21	103.05	250	100.00	400	100.00	247.96	99.18	410.33	102.58
250	500	250.5	100.20	500.85	100.17	249.17	99.67	497.83	99.57	250	100.00	500	100.00	250.05	100.02	496.87	99.37
Mean (%)			100.44		99.71		99.97		100.24		99.47		99.71		99.92		99.71
S.D			1.704		1.560		1.032		2.156		1.504		1.785		0.930		2.559

**Table 7:** Analysis results for the prediction of MLT and PNH in the validation set by the proposed multivariate calibration methods.

Method		GA-ANN				PCA-ANN				WT_ANN				GA-PLS			
MLT	PNH	MLT		PNH		MLT		PNH		MLT		PNH		MLT		PNH	
True(μg mL^−1^)		Found (μg mL^−1^)	R%	Found (μg mL^−1^)	R%	Found (μg mL^−1^)	R%	Found (μg mL^−1^)	R%	Found (μg mL^−1^)	R%	Found (μg mL^−1^)	R%	Found (μg mL^−1^)	R%	Found (μg mL^−1^)	R%
200	100	200.22	100.11	98.36	98.36	199.49	99.75	100.25	100.25	200	100.00	103.84	103.84	200.25	100.13	97.61	97.61
200	200	200.14	99.47	200.18	100.09	199.41	99.71	202.73	101.37	201.2	100.6	200	100.00	200.07	100.04	198.87	99.44
200	300	198.94	97.8	306.35	102.12	200.32	100.16	306.4	102.13	203.42	101.71	300	100.00	200.14	100.07	311.22	103.74
200	400	200.41	100.2	400.61	100.15	200.32	100.16	394.15	98.54	200	100.00	400	100.00	200.76	100.38	394.63	98.66
200	500	200.64	100.32	498.52	99.7	201.03	100.52	496.92	99.38	200	100.00	500	100.00	201.49	100.75	497.9	99.58
300	100	299.94	99.98	99.97	99.97	299.34	99.78	94.74	94.74	300	100.00	96.23	96.23	300.46	100.15	94.59	94.59
300	200	299.42	99.81	200.74	100.37	300.05	100.02	205.86	102.93	300	100.00	199.22	99.61	300.65	100.22	204.36	102.18
300	300	300.09	99.24	303.71	101.24	298.22	99.41	310.37	103.46	302.38	100.79	300	100.00	299.35	99.78	308.28	102.76
300	400	298.75	99.31	402.44	100.61	300.29	100.1	404.06	101.02	300.81	100.27	418.39	104.60	300.47	100.16	404.09	101.02
300	500	300.28	100.09	478.05	95.61	295.75	98.58	487.29	97.46	300	100.00	500	100.00	297.28	99.09	485.16	97.03
Mean (%)			99.63		99.82		99.82		100.13		100.34		100.43		100.75		99.66
S.D			0.747		1.773		0.534		2.678		0.562		2.320		0.425		2.835

**Table 8:** Analysis of MLT and PNH in their bulk drugs and commercial tablets by the proposed multivariate calibration methods and reference method.

		Method	GA_ANN	PC_ANN	WT_ANN	GA-PLS	Reference method ^c^
Bulk drug	MLT	Mean ± SD ^a^	99.63 ± 0.747 (10)	99.82 ± 0.533 (10)	100.34 ± 0.562 (10)	100.75 ± 0.425 (10)	99.96 ± 0.526 (10)
		t test ^b^	1.128 (2.101)	0.591 (2.101)	1.553 (2.101)	0.546 (2.101)	
		F ^b^	2.019 (3.179)	1.030(3.179)	1.143 (3.179)	1.530 (3.179)	
	PNH	Mean ± SD ^a^	99.82 ± 1.773 (10)	100.13 ± 2.678 (10)	100.43 ± 2.320 (10)	99.66 ± 2.835 (10)	99.52 ± 3.076 (10)
		t test ^b^	0.267 (2.101)	0.470 (2.101)	0.744 (2.101)	0.105 (2.101)	
		F ^b^	3.007 (3.179)	1.319 (3.179)	1.757(3.179)	1.175 (3.179)	
Commercial tablets	MLT	Mean ± SD ^a^	97.80 ± 0.889 (6)	98.094 ± 0.486 (6)	97.83 ± 0.765 (6)	97.96 ± 0.0.573 (6)	97.20 ± 0.482
		t test ^b^	1.466 (2.228)	3.212 * (2.228)	1.721 (2.228)	2.512 * (2.228)	
		F ^b^	3.402 (5.050)	1.017 (5.050)	2.519 (5.050)	1.412 (5.050)	
	PNH	Mean ± SD ^a^	100.36 ± 2.944 (5)	105.21 ± 6.188 (5)	103.95 ± 4.781 (5)	99.28 ± 5.965 (5)	103.20 ± 6.20
		t test ^b^	0.925 (2.306)	0.513 (2.306)	0.213 (2.306)	1.019 (2.306)	
		F ^b^	4.437 (6.388)	1.004 (6.388)	1.682 (6.388)	1.081 (6.388)	

^a^ Figures in parentheses are the number of determinations. ^b^ Figures in parentheses are theoretical values for *t-* and *F*- at confidence level of 95%. ^c^ [36]; * Statistically different.
